# Prospects and Aspirations for Workforce Training and Education in Social Prescribing

**DOI:** 10.3390/ijerph20166549

**Published:** 2023-08-09

**Authors:** Abraham Makanjuola, Mary Lynch, Llinos Haf Spencer, Rhiannon Tudor Edwards

**Affiliations:** 1 Centre for Health Economics and Medicines Evaluation (CHEME), College of Health and Behavioural Sciences, Bangor University, Bangor LL57 2PZ, UK; l.spencer@bangor.ac.uk (L.H.S.); r.t.edwards@bangor.ac.uk (R.T.E.); 2 Royal College of Surgeons, 123 St. Stephen’s Green, D02 YN77 Dublin, Ireland; maryalynch@rcsi.com

**Keywords:** social prescribing, link workers, contingent valuation, skills, training

## Abstract

Background: A social prescribing (SP) link worker (LW) is responsible for enabling and supporting individuals, by assessing their personal goals and co-producing solutions to make use of appropriate local non-clinical resources or interventions. As an emerging new role, LWs are not regulated by professional bodies associated with SP. Therefore, currently there is no standardised training for LWs who are from varied backgrounds. As such, LWs have varying knowledge about how to deal with individuals with complex needs, which can impact on their decision-making capabilities to seek solutions and navigate complex systems. The purpose of the research was to explore LWs’ level of education, past and current training requirements as well as elicit how much LWs were willing to pay (WTP) to access and undertake training to improve their skill set. Methods: A rigorous mixed method research design was employed which included semi-structured interviews with key stakeholders and quantitative questionnaires including contingent valuation (CV) questions to a population of LWs across Wales from March to June 2020. Qualitative interviews with key stakeholders who commission and deliver social prescribing interventions employing LWs identified perceived link worker qualities and requirements for LW roles. Purposive sampling was used to identify and select individuals that have experience in managing LWs. Due to the COVID-19 pandemic, interviews were carried out exclusively online. LWs self-selected to complete the online questionnaires. Questionnaires gathered data on LW qualifications and demographic information with the CV questions gathering data on the value LW placed on accessing training in SP. Thematic narrative analysis was applied to interpret the data from the semi-structured interviews. Descriptive frequency analysis was conducted on the quantitative data generated from the online questionnaire. Findings: SP coordinators (*n* = 6) reported that ‘personal skills’ are the most essential skills required by LWs in SP intervention. Training is available for LWs; however, the training undertaken varies depending on the type of intervention delivered, with 70% of LWs previously undertaking training to facilitate their development as an LW. The results from the contingent valuation questionnaire (*n* = 54) indicated that 100% of the respondents would avail of training. LWs were asked how much they were willing to pay as a single payment for professional training; on average, LWs were WTP GBP 58 from their personal funds to access training and the associated benefits to enhance their skills and knowledge. Interpretation: The semi-structured interviews conducted with the key SP stakeholders yielded rich information and novel insight into LW training. External funding for the salary of the LW is an obstacle for LW development through training. In addition, the questionnaire results regarding stated preference techniques demonstrate that LWs place value on their professional development and would be willing to spend their own money on training to improve their knowledge and skills.

## 1. Introduction

Social prescribing (SP) is a non-clinical intervention which involves connecting citizens to community supports to better manage their health and wellbeing and to improve outcomes [1]. SP is a means of enabling General Practitioners (GPs), nurses and other primary care professionals to refer individuals to a range of local, non-clinical services [2]. A link worker (LW) is responsible for enabling and supporting an individual to assess their requirements, co-producing solutions for them and making use of appropriate local resources [3]. Individuals who chose to use SP interventions require LWs to signpost them to various SP community activities. Taking a holistic approach in tailoring SP options to meet the requirements of individuals necessitates LWs to form relationships across a spectrum of individuals, organisations and community groups within society. LWs assist patients in managing chronic illnesses by signposting patients to community healthcare services that they were previously unaware of to improve wellbeing [4]. Tailoring patient care in SP promotes long-term wellbeing via prevention and the delaying of long-term conditions [5]. SP was also highlighted as one of the ten high impact actions outlined to ease GP workload [6]. SP intervention takes a number of different forms to seek solutions to meet an individual’s requirements. Some of the different types of SP intervention include arts-based prescription, exercise referral and green social prescribing [7,8,9]. This requires LWs to have a range of personal attributes along with good communication, knowledge and skills to navigate complex systems to develop social capital and the wellbeing of individuals and communities. As an emerging new role, LWs are not regulated by professional bodies and there is no consistent training for LWs who are joining the practice of SP from varied backgrounds. As such, LWs have varying knowledge about how to deal with individuals with complex requirements, which can impact on their decision-making capabilities to seek solutions and navigate complex systems. Therefore, this can impact on their decision-making capabilities and influence the training which may be necessary to build confidence to seek solutions [10]. Training to support LWs to manage complex case referral is essential [11]. The LW title can vary depending on the type of SP intervention; these titles include community connector, wellbeing advisor and social prescriber [12]. Although the titles can vary, there is overlap in the fundamental skills required for the role among the different titles. Recent evidence has assisted in creating internationally recognised theoretical and operational definitions of social prescribing which should be integrated into future social prescribing research and policy development [13]. There is some supporting evidence [14] indicating that LWs can provide unqualified specialist support to vulnerable individuals using SP interventions. Currently, there is no specified training for LWs; however, it is advised that new LWs working within a primary care network (PCN) complete the mandatory Health Education England (HEE) e-learning programme, and they are advised to avail of peer support networking opportunities [15]. However, not all LWs work within a PCN. Integrated within this study are key contingent valuation (CV) questions to understand LWs’ choices and preferences to undertake additional training to improve their skills and abilities to carry out their role. The CV approach using willingness to pay (WTP) captures the value estimates that LWs place on the true value of accessing and undertaking training [16].

This study was funded by the Knowledge Economy Skills Scholarship (KESS 2) funded by the Welsh Government along with partner organisation Conwy Council. The purpose of this collaborative project was to examine the requirements of LWs who were delivering more SP interventions within the Conwy Council area as well as throughout Wales. The aim of this study was to explore LWs’ level of education, past and current training requirements as well as elicit LW value estimates to undertake additional training and willingness to pay (WTP) to access training.

## 2. Methods

The NHS in England has supported SP integration into primary care and community settings; however, that has not evolved in Wales as there are many commissioners. The Wales School for Social Prescribing Research in collaboration with Public Health Wales and Health and Care Research Wales are driving forward the agenda for SP in Wales. This study was conducted in Wales, UK, with data collected from March to June 2020. This study applied a mixed method research design which included semi-structured interviews with key stakeholders and quantitative questionnaires including contingent valuation (CV) questions. Qualitative interviews were conducted with key stakeholders who commission and deliver social prescribing interventions employing LWs to distinguish perceived link worker qualities and requirements for LWs’ employment in SP programmes. These considered LW characteristics were integrated into the questionnaire that was developed to gather data on LW qualifications and demographic information, and it included CV questions based on choice and preferences in accessing training requirements. CV is an appraisal technique used to secure public participation as the technique elicits information on attitudes, motivations, preferences and willingness to pay [17]. Using the exponential payment ladder, valuation estimates were elicited from LW on the willingness to pay (WTP) for training to improve skills and knowledge.

Qualitative interviews were undertaken with key stakeholders who commission and manage SP interventions in the community. The purpose of the semi-structured interviews was to gain insight into the important skills and training that the SP intervention commissioners consider necessary for LWs to carry out effective delivery of SP programmes. Following the semi-structured interviews, an online stated preference techniques questionnaire containing contingent valuation (CV) questions was shared among a Welsh network of LWs who deliver SP programmes in Wales, UK.

### 2.1. Qualitative Interviews with Key Stakeholders

The interviewees were identified as key stakeholders in SP in Wales by regional commissioners of SP services. In order to be considered for an interview, participants had to manage a team of LWs and coordinate the delivery of SP on a local or regional level. The primary objective of the semi-structured interviews was to gain insight into the skills and training available for LWs in SP from a managerial perspective. In addition, the social prescribing coordinators were asked to share their perspectives on what kind of training is valued for their role in commissioning SP interventions to members of the community living with complex care demands. Semi-structured interviews are in-depth interviews seen in qualitative methodology, which are often used by a range of healthcare professionals in research [18]. Semi-structured interviews follow the format of the interviewer asking predetermined core questions on a specific point of inquiry.

Follow-up questions based upon the response of the interviewee were asked to provide further context. Given the richness of semi-structured interview data, semi-structured interviews were recorded to ensure all verbal data were collected. Recordings allow for verbatim quotes to be transcribed and analysed, and then thematic analysis was employed in this study to contextualise the findings and to draw key points of further discussion [19]. Thematic analysis within semi-structured interviews allows for the researchers to draw parallels between the varying perspectives of different interviewees within the sample [20].

### 2.2. Semi-Structured Interviews

The semi-structured interviews comprised of 15 questions prepared for the purpose of this study. All the questions in the interview schedule fell into one of the following categories: social prescribing interventions; demographic information; information about the social prescriber coordinator role; funding; qualifications, skills and training; and peer support. The themes of the semi-structured interviews were derived from the key findings from in-depth interrogation of the literature on LW training and skills.

To target key commissioners and SP coordinators to participate in the project, a focused recruitment advert was circulated to potential commissioner respondents through the Wales School for Social Prescribing Research (WSSPR) to attract interview participants. Commissioners were interviewed as part of the questionnaire development phase, and six SP coordinators based across Wales, UK, responded to the advert and were interviewed between March and June 2020. As the six SP coordinators gave similar responses, data saturation was reached by the sixth interview [21]. Given the COVID-19 pandemic, the interviews were conducted over the video communications platform Zoom [22]. Participants received information sheets and consent forms electronically; however, given that the interviews took place over the internet, verbal consent was taken at the beginning of the interview, and the interviews were audio recorded on the videoconferencing software Zoom. The answers to each of the interview questions asked were transcribed into Excel [23] verbatim. Thematic analysis focusses on the generation of themes from the dataset. Within this study, thematic framework analysis was used to analyse the qualitative data. The first step within this framework is familiarisation with the content; the second step is coding the data; the third step is generating the themes; the fourth step is reviewing the themes within the research group; and the fifth step is naming and defining the themes [24].

Four themes were interpreted from the patterns found which were important to describe and interpret the topic of LWs linked with the perceived values and attributes of LWs. The thematic analysis followed a staged process by reading and rereading the entire dataset. Initial thematic codes were generated by AM, on the basis of pre-existing knowledge within the field of SP. The identified four overarching themes were reviewed by ML and LHS: funding, the SP coordinator role, training for LWs and peer support. These agreed themes were finalised by reviewing and revising against the initial codes to check for consistency. The theoretical framework findings are presented in the results section.

### 2.3. Quantitative Survey

Due to the COVID-19 pandemic, the questionnaire, which was originally developed in a paper format, was placed on KoBoToolbox, an open source digital questionnaire platform during May and June 2020 [25]. This platform was selected as it did not collect the IP addresses of respondents, which would help maintain anonymity in compliance with the General Data Protection Regulation (GDPR) which ensures the safeguarding of personal data in healthcare research [26]. The questions elicited information regarding personal characteristics, LW experience, education, salary, disposable income and training requirements. Descriptive frequency analysis was conducted on the quantitative data.

The questionnaire contained contingent valuation (CV) questions to examine the value that respondents place on accessing training and to elicit respondents’ willingness to pay (WTP) for goods not normally traded in the open market [27]. These non-market valuation techniques were used in order to ascertain choice and preference to undertake additional training as well as value the benefits of increased access to training for LWs [28]. The value assigned to training was elicited via an exponential payment ladder to assess the value that LWs place on the benefits of professional development training and networking. Following the valuation exercise, respondents were asked follow-up questions to understand the choices and preferences of respondents and the value placed on accessing training along with the constraints and rationale for the responses and valuation estimates.

## 3. Results

### 3.1. Qualitative Findings

There were (*n* = 6) SP coordinators interviewed for this study with 67% identifying as female and 33% as male. The qualitative interview data were analysed within four themes based on the questions and responses provided [24], which are as follows: social prescribing role, funding, peer support and training.

### 3.2. The Social Prescribing Coordinator Role

This theme comprises of insights into the job roles of the interviewees, their experience in the field and the type of SP activities delivered through their organisation. SP coordinators were asked to outline the SP activities delivered through their organisation to have a greater knowledge of the breadth and depth of SP intervention. The findings indicate that SP coordinators commission and manage the delivery of community-based SP interventions. These activities include gardening, arts and cultural activities, life coaching, social groups and walking groups.

*“Our project is specifically linked to GPs surgeries…”*

*“Some are based in Primary Care…”*

*(Interviewee 6)*

A range of job titles were reported by SP coordinators, which include Chief Officer, Exercise Referral Coordinator, Community Wellbeing Team Leader, Programme Director, Business Development Officer and Project Lead. For example:

*“My formal job title is Programme director”*

*(Interviewee 3)*

To understand the level of experience that SP coordinators had in their roles, SP coordinators were asked to report their length of tenure in their current roles. The results show that SP coordinators were employed in their roles on average for 4 years and up to 9 years, and the amount of experience in the area of SP delivery ranged between 3 and 20 years.

*“I have been doing that [Social Prescribing] since about 2003 so about 16 years”*

*(Interviewee 1)*

### 3.3. Funding for Social Prescribing Roles

This theme outlines the sources of funding for SP projects which include the staffing costs of LWs and SP coordinators. To understand the size of the teams required to deliver SP programmes, SP coordinators were asked to indicate the number of LWs that they managed. SP coordinators reported an average of 35 LWs within a team, with a range of 2 up to 150 LWs.

To understand the contractual obligations SP coordinators, SP coordinators were asked to state the duration of their contracts. Half of the SP coordinators interviewed were on fixed-term contracts, and the remainder were on permanent contracts. SP coordinators were asked to state the nature of their funding; half of the SP coordinators were paid via core funding and the remainder were supported through grant funding. Funding for SP initiatives within Wales is supported through various sources including the Welsh Government which cascades down to Public Health Wales, Welsh Wellbeing Fund, Service Level Agreements with local authorities and Welsh NHS Trusts. In addition, charitable funds such as the Big Lottery Fund also support SP initiatives [29].

*“Yes, the project is fully funded by the Welsh Government. The salaries and all the related costs”*

*(Interviewee 2)*

All the LWs managed by the SP coordinators receive their salary from external funding; one interviewee cited that this is an obstacle for LW development.

*“What I would like to see is, that the grant funding becomes core funding so that people have more security in their roles and will therefore develop in their roles”*

*(Interviewee 3)*

### 3.4. Peer Support for Link Workers

This theme takes into account the type of peer support that is available for LWs to carry out their role. All the SP coordinators reported that the LWs they supervise had access to peer support. The findings indicated that most of the SP coordinators reported that ‘intra-team’ peer support is available, whereby LWs can discuss challenges and obstacles with the other LWs in their team and with their line manager. In addition, around a third of the SP coordinators reported that the LWs on their team received peer support from the social prescribing network (SPN). Less than a quarter of the SP coordinators reported that peer support was available through local authority initiatives, i.e., counselling.

*“We have regular supervisions, and we have team meetings on a monthly basis”*

*(Interviewee 4)*

*“Our closest GP surgery we’ve been working with for 25 years on Social Prescribing we’ve always been here as a charity offering Social Prescribing it just didn’t have the buzz name”*

*(Interviewee 6)*

### 3.5. Training for Link Workers and Social Prescribing Coordinators

This theme examined the type of training that LWs and SP coordinators have undertaken as well as the SP coordinators’ views pertaining to essential skills of LWs. To understand which skills they perceived as necessary for LWs delivering SP interventions, the SP coordinators were asked which skills they felt were most necessary to carry out the LW role. The findings indicate that communication and listening skills, adaptability along with management and organisational skills were amongst the most essential skills that LWs require.

To identify any gaps within training requirements, SP coordinators were asked to state the training provided for the LWs within their teams. SP coordinators cited a vast range of training, i.e., the Future Generations and Social Services Acts and dementia awareness training [30,31].

*“We do the [training] around the Future Generations Act… We try to encourage them to do Dementia awareness training… we’ve got a team that like doing different things”*

*(Interviewee 1)*

Interviewee 2 reported that bespoke SP training was available via a module delivered by a local university and mental health charity.

*“I have links with [a local] University and they have a Link Worker course and they gave me 2 free courses … and then [our research funder] developed its own course for the Link Workers”*

*(Interviewee 2)*

Interviewee 3 reported SP training at [a local] University.

*“Training [at a local University] is available and learning on the job”*

*(Interviewee 3)*

Interviewee 4 reported Health and Safety training, the Prince 2 management program and asset-based community development training.

*“The ABCD which is the Asset Based Community Development training is one of the main ones [training] staff have been required to undertake… and also a project management qualification such as the Prince 2”*

*(Interviewee 4)*

Interviewee 5 reported motivational interview training, Public Health Wales’s online course ‘Making every contact count’ and the safeguarding vulnerable adults course.

*“The referral staff [some of them] have had motivational interview training and everyone has making every contact count from Public Health Wales… they also need to have completed safeguarding training of vulnerable adults…”*

*(Interviewee 5)*

Interviewee 6 reported training on the online SP platform Elemental, motivational interview training and admin training.

*“We’ve got a training for Elemental [software] training next week”*

*(Interviewee 6)*

When asked which types of training would benefit an individual carrying out the role of SP coordinator, most participants responded with some form of leadership and management training, such as the Prince 2 management program and asset-based community development training. In addition, it was noted that essential training included health and safety training, public health training and level 3 exercise referral training to effectively carry out their role.

*“The field of Social Prescribing is so broad… there’s something missing in between a vocational qualification and making every contact count, just some training around how to ascertain what is important to people, what would be of benefit and make the links of what is going on locally… and deal with any barriers that come up”*

*(Interviewee 5)*

### 3.6. Quantitative Survey Findings

LWs (*n* = 54) completed the questionnaire with 87% identifying as female and 13% as male. The results indicate that 50% of respondents stated their job title as a community connector, with a further 15% of respondents using the title of wellbeing officer as shown in Table 1. In addition, 13% had the title of link worker, and 11% of respondents had the titles of local asset coordinator and exercise instructor.

LWs were asked to report the highest level of education they had attained; 23% of LWs had attained a master’s, and a further 43% of LWs indicated they had attained an undergraduate degree, as shown in Table 2. In addition, 30% of LWs had attained A Levels or a Business and Technology Education Council (BTEC) qualification, with 4% having gained General Certificate of Secondary Education (GCSE). When asked about professional training to encourage growth in the LW role, 70% of LWs indicated that they had completed training to develop in their role, and 30% had not completed any professional training in their current role.

### 3.7. Contingent Valuation Results

All LWs were asked to indicate if training was available free of charge which would enhance their skills in SP, had modules on new approaches to the role and access to a network of other LWs; 100% (*n* = 54) of LWs would avail of the free training. To understand LWs’ ability to pay for additional training, all respondents were asked to indicate their hourly rate of pay. LWs (*n* = 53) reported their hourly rates of pay which ranged from GBP 8.75 to GBP 29.38. On average, LWs’ hourly rate of pay is GBP 14.20, which is the equivalent of GBP 24,995 per annum for an LW working full time for 35 h per week. Then, LWs were asked if the aforementioned training was no longer available free of charge to indicate the value that they would place on accessing this training via an exponential payment ladder card, with estimates ranging from GBP 0 to GBP 600 to participate in training. LWs were asked to indicate the maximum they were willing to pay (WTP) from their own pocket to undertake this training, taking into account what they could realistically afford to pay given their current financial situation. LWs indicated an average WTP of GBP 58 to access training to develop their skills, and this was the value they placed on accessing training, as shown in Table 3.

The LWs reported an average yearly salary of GBP 24,995. To understand the reasons that LWs had selected the valuation estimates to undertake training, LWs were asked about their WTP choices for participating in training. All LWs (*n* = 54) outlined their rationale, with 19% of LWs reporting that the valuation selected reflected the value they placed on accessing and participating in the training. In addition, 48% of LWs reported that they do not believe that they should pay for training, with a further 33% of LWs reporting that they could not afford to pay out-of-pocket expenses to undertake the training. Finally, to understand the impact of income on their ability to engage with additional training requirements, LWs were asked to consider their current financial situations in relation to the cost of living. All LWs (*n* = 54) responded to this question with 6% reporting to be comfortable and 26% indicating they could manage without much difficulty as shown in Table 4. However, 61% of LWs reported that they have to be careful with money, with a further 7% stating it was a strain to get from week to week. The findings would suggest that LWs place value on accessing training to improve their skills, earn on average GBP 14.20 per hour and that the impact of the cost of living affects 68% of LWs in terms of their ability to support additional training requirements from their disposable income.

## 4. Discussion

SP connects individuals to community support by means of referral to an SP LW. Following referral, a “What matters?” conversation occurs to co-produce solutions with individuals which are focused on empowering and connecting individuals to local community assets. This active involvement within the community assists in accessing local social and community supports such as information advice, social and physical activities and lifestyle behaviour support which assist in improving health and wellbeing outcomes [32].

The main aim of this study was to present the findings from an exploratory study examining the emerging new role of LWs, to explore LWs’ level of education, along with the motivation to engage with training opportunities to improve their skill set. In relation to qualifications, it was found that LWs held a variety of qualifications, from secondary school qualifications only to postgraduate master’s qualifications. The LWs had an average of around 5 years of experience (ranging from 1 year to 29 years) in connecting individuals with local community assets to improve health and wellbeing outcomes. Some LWs were new to the role, and some had been doing similar work for up to 29 years.

The diversity of the role and job titles makes it difficult to articulate what social prescribers/community link workers/community connectors do, and therefore, their role is not well known to primary care, to government teams or the public [33]. These challenges are compounded by the range of employers (local authorities, health boards and third sector organisations) with different terms and conditions, different commissioning arrangements and local variation.

The sustainability of the role is also challenging due to fixed-term funding, contingent on specific grants from local or national government or third sector partners [34]. Most SP coordinators in this study reported that ‘Personal skills’ are the most essential skills required by LWs in SP intervention. Personal skills refer to communicational skills, empathy and the ability to listen to the requirements of the service user [35]. Training is available for LWs; however, the training undertaken varies depending on the type of intervention delivered by the LW. Evidence on social prescribing is gathering momentum, with findings suggesting that social prescribing is increasing linked with social inequalities, increased healthcare resource use and building on responsibility for self and personal care [36]. However, it is suggested that social prescribing should take a significant holistic approach which integrates evolution to support and enhance primary care using community assets.

This study highlights the potential to look at service standards, roles, career pathways and to consider accreditation of prior learning, qualifications and apprenticeships of link workers in social prescribing. This study also builds upon the work of the 2019 National Association of Link Workers report [35] by demonstrating that LWs place a value (GBP 58) on training to aid in their professional development. It is necessary to determine who pays for LWs continuing professional development. The majority of LWs indicated that they did not believe it is their responsibility to pay for their training and that they cannot afford to self-fund. Furthermore, concerning the cost of living in relation to income, the majority of LWs indicated that they have to exercise prudence with their disposable incomes given that the average salary for the LW role was GBP 24,495 in 2020, which was below the yearly cost of living in the UK of GBP 30,285 per annum [37].

### 4.1. Strengths

This innovative mixed method social prescribing research contributed to the growing knowledge base around LW roles and training requirements. This study also provided new evidence to support the development of the LW role.

The findings of this research have highlighted the gap in the development of a structured training package of modules for LWs working in the field of social prescribing in Wales.

### 4.2. Limitations

The number of total LWs working across Wales is unknown; therefore, it is unclear to what extent the questionnaire sample was representative of the number of LWs employed in Wales. In addition, the authors recognise that the level of rich data is a limitation due to the number of SP coordinators who responded to the advert which was circulated through the WSSPR network during the initial consultation phase.

The LW questionnaire could have benefitted from questions that asked LWs about who paid for their training to date and which types of training would be beneficial for their future.

Due to COVID-19 restrictions, videoconferencing had to be used for the interviews, and this meant that it was more difficult to build rapport between the interviewer and interviewees. Being face to face allows for better exchange of information as both speaker and listener are able to see and interpret body language and facial expressions [38].

## 5. Recommendations

- For future social prescribing interventions, training requirements should be included in the funding bids to ensure that link workers receive adequate training to carry out their role.
- Training for social prescribing link workers should be made available in their own local areas.
- The findings are based on the responses of the (*n* = 52) LWs who completed the questionnaires to state that they are motivated to undertake training to improve their skills and knowledge. This research study demonstrates LWs’ attitudes, preferences and values indicated by their WTP for future training. The results also indicate that there is a benefits transfer on the average WTP estimate of GBP 58 from this study, suggesting a proposed change to the provision of proposed training for LWs.

## 6. Conclusions

In summary, link workers are willing to pay GBP 58 of their own disposable income to enhance their skills as social prescribers. As the scope of their work is enormous, link workers would benefit from training to enhance what they believe is the core principle of their work, which is personal skills such as listening and responding to the unique individual requirements of their clients, which may be complex.

## Figures and Tables

**Table 1:** Different job titles of the link worker role.

Job Titles	*n*	Percentage
Community Connector	27	50%
Wellbeing Officer	8	15%
Link Worker	7	13%
Exercise Instructor	6	11%
Local Asset Coordinator	6	11%
**Total**	54	100%

**Table 2:** Link worker education.

Highest Level of Education	Percentage
General Certificate of Secondary Education (GCSE)	4%
A Level/Business and Technology Education Council (BTEC)	30%
Undergraduate Degree	43%
Master’s Degree	23%
**Total**	100%

**Table 3:** Respondents’ WTP.

Payment Ladder Bid Vectors	*n*	Respondents’ WTP Percentage
GBP 0	21	39%
GBP 10	6	11%
GBP 50	15	28%
GBP 100	3	5%
GBP 150	7	13%
GBP 350	1	2%
GBP 600	1	2%
**Total**	54	100%
**Average WTP**		GBP 58
**Median WTP**		GBP 30

**Table 4:** Respondents’ perception of cost of living.

Cost of Living	*n*	Percentage
I have to be careful about money	33	61%
I am able to manage without much difficulty	14	26%
I find it a strain to get from week to week	4	7%
I am quite comfortably off	3	6%
**Total**	54	100%

## Data Availability

The final dataset will only be available to the study investigators and the advisory team. Informed consent was obtained from all subjects involved in the study.
